# Gun-shot Wound

**Published:** 1843-08

**Authors:** J. H. Thompson

**Affiliations:** Salem, N. J.


*Gun-shot Wound. Extensive laceration of the Brain, without loss of consciousness, or impairment of mind.* By J. H. Thompson, M.D., of Salem, N. J.—*Monday, February 13th*, 1843. Mr. Wm. L. Johnson, ætat. 22, in attempting to shoot a bullock on his father's farm, which is about two miles from this town, was wounded by the bursting of the gun, a fragment of which struck him in the median line at the root of the nose. I saw him in about twenty-five minutes after the occurrence. The hemorrhage, which was said to have been profuse, had nearly ceased. At the moment of the accident he was prevented from falling by a person near him. He then walked into the house, a distance of about forty yards. I found him, although suffering agonizing pain, in the full possession of his mental faculties, which he had not indeed for a moment lost. Considerable tumefaction had occurred in the injured parts. The wound resulting from the accident commenced a little above the transverse suture, and extended directly down the middle of the nose. It was three quarters of an inch in length, by one quarter in breadth. The nasal bones were completely demolished, the frontal sinuses were laid open, and the upper turbinated bone was torn off. This much of the injury could be distinctly seen. The missile after entering had taken an oblique direction behind the right eye. Its ultimate course could not be accurately traced, owing to the extreme sensibility of the wounded parts; nor indeed was it deemed proper to persevere in the attempt, since it soon became evident that the foreign body had penetrated so deeply as to render it highly probable, that more injury than benefit would result from the search. The right eye protruded more than half an inch beyond its ordinary level. This protrusion was an immediate consequence of the accident, and therefore led to the supposition that the optic nerve was destroyed. The patient referred all his pain to the right eye. The sensibility of this part was so acute, that the slightest touch—even a drop of water falling upon it, brought on spasmodic action of the whole muscular system, and rendered the dressing of the wound a task of great difficulty. Dr. Swing, an experienced practitioner, residing at Sharpstown in this county, was called in consultation with me, and continued to attend the case until its termination. Several loose fragments of bone were removed from the wound, and cold water dressings were applied. It is not necessary to enter further into the treatment than to say, that the strictest antiphlogistic plan was adopted and rigidly pursued. During the first and part of the second day, this amiable and unfortunate young gentleman retained the perfect possession of his senses. He answered all questions readily and with entire consistency—knew his friends—called repeatedly for different members of his family—made use of the most tender and endearing expressions towards them—in short, his mental faculties appeared to be unimpaired. There was no paralysis manifested until a few hours before death, when the *left arm* became motionless. Delirium came on, on Tuesday afternoon, and continued with short intervals until the termination of the case. Suppuration was speedily established; several considerable fragments presented themselves, and were removed. At the second dressing, a small quantity of cerebral substance issued from the wound. Death occurred on Friday morning, four days after the reception of the injury. Permission for a post-mortem examination was granted, on condition that we would make no incisions, nor in any way disfigure the body. We were therefore limited to a mere inspection of the wound. This is to be regretted, since it would have been interesting to have ascertained with precision, the *exact* nature and extent of the injury which had been inflicted. Tumefaction having subsided, the wound now presented a very different appearance. It measured one inch and a half in length, and an inch in breadth. A fissure commencing a little to the right of the median line, extended perpendicularly about two inches up the os frontis. As before mentioned, the frontal sinuses were opened; two or three considerable fragments of the internal table of the os frontis were depressed. The wound was filled with the softened substance of the brain, and with pieces of bone. As well as could be ascertained by the introduction of a finger, all the bones entering into the composition of the orbit, with the exception, perhaps, of those forming the anterior part of the floor, were broken up. The right eye was completely torn loose from all its attachments to the posterior part of the orbit. Through a large opening formed by the fracture of the orbitar plates of the sphenoid and os frontis, the finger could be passed into the skull. As far as it could be reached in this way, the brain was in a pulpy condition. No foreign body could be detected. A probe, however, at length made it apparent. It proved to be the whole breech-pin of the gun. The large end, or that part which is screwed into the barrel, was over or upon the petrous portion of the temporal bone, the small end was near the opening which its passage had made in the bones of the orbit. The weight of this piece of iron (which is now in my possession) is two ounces; it is two inches and three quarters in length. Its extraction was attended with considerable difficulty. It could not in fact be removed until we had taken away, with strong forceps, the depressed portions of the os frontis.

Here was a most extensive laceration of the brain from a foreign body which remained imbedded in it, large fragments of bone were depressed, and in all probability spiculæ were driven into the cerebrum, yet none of the ordinary signs of such an injury were present. There was no loss of consciousness—no symptoms of compression—no paralysis until a few hours before death,—and until delirium came on as a consequence of inflammation of the brain, the operations of the mind were unimpaired. It is presumed that in so dreadful an injury as this, the case would necessarily be considered hopeless, although Larrey, Dupuytren, and indeed all sugeons relate instances of recovery after more or less extensive injury of the brain. There are upon record at least two examples of wounds from precisely the same cause as the one which came under my observation. The first is related by Dr. Rogers, (Vide *Am. Journal of Med. Sci.*, for 1828. The account is extracted from *Med. Chirurg. Trans*). In this case the breech-pin of the gun remained in the left hemisphere of the brain for twenty-seven days. The patient recovered with the loss of the sight of the left eye, and with his mental faculties unimpaired. In the second case both hemispheres were wounded to the depth of an inch and a half by the breech-pin of a gun, which struck the subject of the injury in the middle line of the frontal bone. (Vide *Am. Journal of Med. Sci.*, for 1830, where the account is taken from the *Edinburg Medical and Surgical Journal*). In this case also, the patient perfectly recovered "without the slightest alteration in mental power."—*American Journal.*

